# Graphene–DNAzyme junctions: a platform for direct metal ion detection with ultrahigh sensitivity†
†Electronic supplementary information (ESI) available. See DOI: 10.1039/c4sc03612c



**DOI:** 10.1039/c4sc03612c

**Published:** 2015-02-05

**Authors:** Li Gao, Le-Le Li, Xiaolong Wang, Peiwen Wu, Yang Cao, Bo Liang, Xin Li, Yuanwei Lin, Yi Lu, Xuefeng Guo

**Affiliations:** a Center for Nanochemistry , Beijing National Laboratory for Molecular Sciences , State Key Laboratory for Structural Chemistry of Unstable and Stable Species , College of Chemistry and Molecular Engineering , Peking University , Beijing 100871 , P. R. China . Email: guoxf@pku.edu.cn; b Department of Chemistry , University of Illinois at Urbana-Champaign , Urbana , Illinois 61801 , USA . Email: yi-lu@illinois.edu; c Adesso Advanced Materials Wuxi Co., Ltd. , Huihong Industrial Park , 18 Xishi Road, New District , Wuxi , Jiangsu Province 214000 , P. R. China; d Department of Materials Science and Engineering , College of Engineering , Peking University , Beijing 100871 , P. R. China

## Abstract

Many metal ions are present in biology and in the human body in trace amounts. Despite numerous efforts, metal sensors with ultrahigh sensitivity (<a few picomolar) are rarely achieved. Here, we describe a platform method that integrates a Cu^2+^-dependent DNAzyme into graphene–molecule junctions and its application for direct detection of paramagnetic Cu^2+^ with femtomolar sensitivity and high selectivity. Since DNAzymes specific for other metal ions can be obtained through *in vitro* selection, the method demonstrated here can be applied to the detection of a broad range of other metal ions.

## Abstract 2

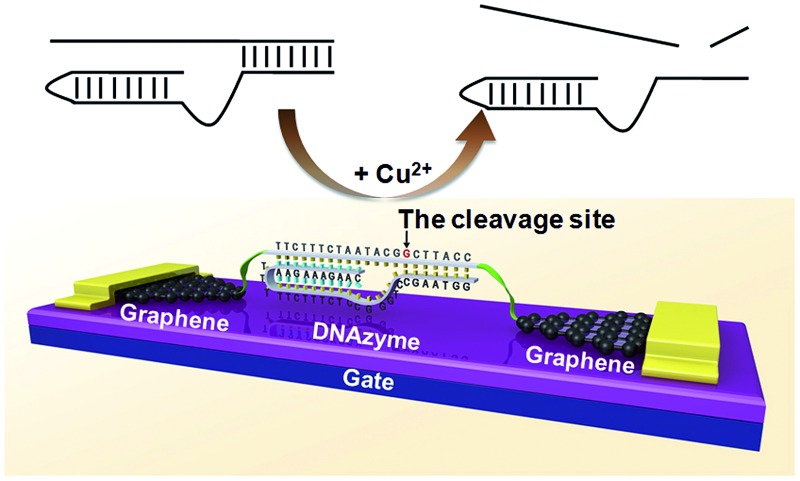
We describe a method of creating graphene–DNAzyme junctions capable of directly detecting paramagnetic Cu^2+^ with femtomolar sensitivity and high selectivity.

## Introduction

**Fig. 1:**
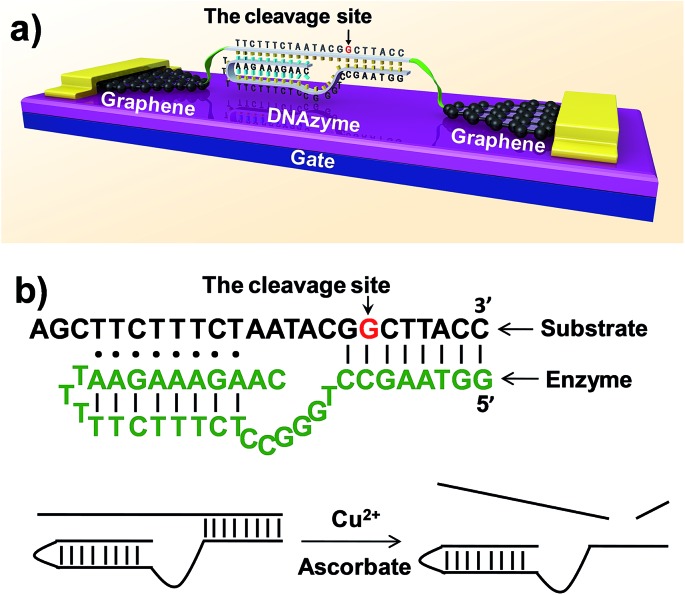
(a) Schematic representation of graphene–DNAzyme junctions. (b) The structure of the Cu^2+^-sensitive DNAzyme and corresponding catalytic activity. The DNA substrate has been functionalized by amines on both ends for molecular connection (see the ESI†). The cleavage site is indicated by an arrow.

The ability to detect chemical and biological species at ultra-low concentrations is important in many areas, ranging from the diagnosis of life-threatening diseases to the detection of biological agents in warfare or terrorist attacks.^1–7^ Here, we describe a method to achieve the direct detection of paramagnetic Cu^2+^ with femtomolar sensitivity and high selectivity by using single-molecule graphene–DNAzyme junctions (Fig. 1). Copper ion is an essential metal ion for many biological functions. Recent studies have shown that bioavailable copper ion in organisms is relatively low. For example, the concentration of free copper ions is said to be ∼10^–21^ and ∼10^–18^ M in *Escherichia coli* and yeast, respectively.^8,9^ This low level of free copper ions is crucial, as increased copper levels are highly toxic, which can cause gastrointestinal disturbance and liver or kidney damage.^10,11^ Therefore, direct copper ion detectors with high sensitivity and selectivity are very useful in understanding its roles in biology. Towards this goal, many fluorescence small-organic-molecule-based Cu^2+^ sensors have been developed based on the changes in their fluorescence intensity upon binding to Cu^2+^ (ref. 12 and references therein). Most of these sensors, however, require the incorporation of a fluorophore into the metal recognition site, using an organic solvent, and cannot reach the sensitivity required for detection. Only a few such sensors demonstrated nanomolar sensitivity with high selectivity and without using an organic solvent.^7,13–22^ An efficient way to overcome these problems is to develop nanomaterial-based electrical biosensors that allow ultrasensitive and direct electrical detection of target analytes in a nondestructive manner.^23,24^ In particular, we are interested in using nanoscale junctions bridged by molecules, such as catalytic DNA or DNAzymes, to build metal sensing platforms offering unique advantages, such as low cost, portability, ultra-high sensitivity, and excellent selectivity.

DNAzymes are DNA-based biocatalysts that have the ability to perform many chemical and biological reactions.^25–27^ Most of these reactions require specific metal ions as cofactors. As a result, a number of highly effective fluorescence, colorimetric, and electrochemical sensors based on DNAzymes have been developed for detecting different metal ions,^2^ such as Pb^2+^,^28–30^ UO_2_^2+^,^22^ Hg^2+^,^31,32^ Cu^2+^,^21^ and others.^33^ Compared to proteins or RNA molecules, DNAzymes are an excellent choice for metal ion detection because of their relatively low cost and high stability towards hydrolysis. In addition, the DNAzymes can still be active even after many cycles of denaturation/renaturation. These properties are ideally suited for electrochemical device engineering and manufacturing. Despite these advantages, DNAzyme-based sensors for ultrasensitive detection of metal ions (less than a few nanomolar) have rarely been achieved. In this study, we aim to demonstrate a new platform for ultrasensitive detection of metal ions by integrating a Cu^2+^-dependent DNA-cleaving DNAzyme into graphene–molecule junctions (Fig. 1). On the basis of the original DNAzyme sequences,^34–36^ we designed a Cu^2+^ electrical sensor consisting of a DNA substrate strand with amines on both ends for the connection to the graphene–molecule junctions, and an enzyme strand that can hybridize to the substrate strand through two base-pairing regions (Fig. 1). The 5′-portion of the enzyme binds the substrate *via* Watson–Crick base pairs and the 3′-region through the formation of a DNA triplex. Initially, the complex is conductive through π–π stacking.^37^ In the presence of Cu^2+^, the substrate is cut at the cleavage site (the deoxyguanosine shown in red and indicated by an arrow in Fig. 1). Because the melting temperatures of the two cleaved fragments are lower than room temperature, the fragments are released (Fig. S1†), leading to the breakage of the junctions, and thus a decrease in device conductance. In addition to employing highly selective DNAzymes, a unique feature of our design is the use of graphene–molecule junctions that consist of one or a small collection of molecules as conductive elements.^38,39^ This combination can lead to ultrasensitive functional electronic devices and new classes of chemo/biosensors with single-molecule sensitivity.^40–49^

## Results and discussion

The graphene–DNAzyme devices were built using a dash-line lithographic (DLL) method described in detail elsewhere.^39^ The key feature of this DLL technique is the ability to produce nanogapped graphene point contact arrays that can be functionalized by carboxylic acid on each side. These point contacts react with conductive molecules derivatized with amines to form molecular devices in high yields. In addition to this feature, another important advantage of this technique is that the contacts made by covalent amide bond formation are robust and thus can tolerate various kinds of chemical treatments. In conjunction with the electrical properties of graphene electrodes, the ease of device fabrication and the device stability place the graphene–molecule junctions as a promising testbed for molecular electronics.^50^

**Fig. 2:**
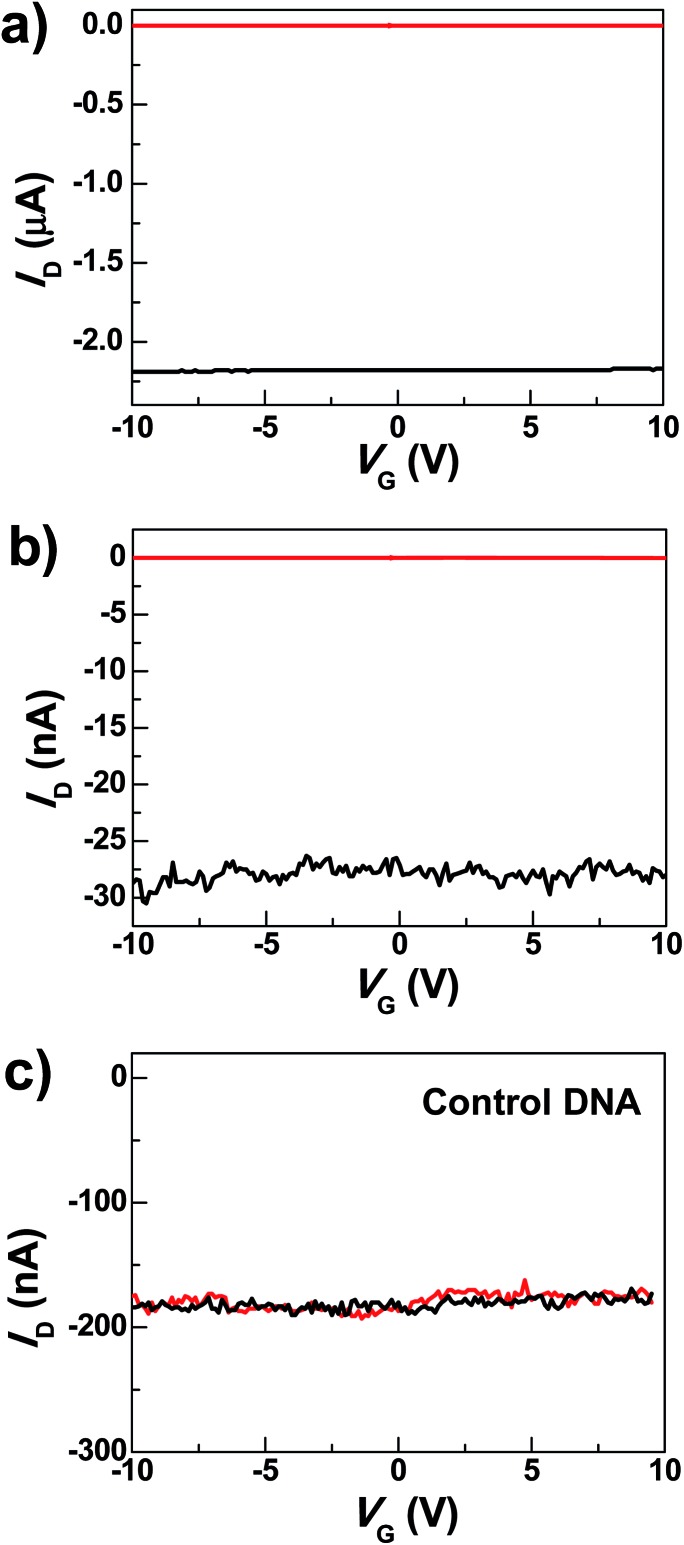
(a) *I*–*V* curves of a graphene device before (black) and after (red) cutting. (b) Device characteristics of a representative DNAzyme-reconnected device after DNA connection (black) and further Cu^2+^ treatments (0.5 nM, 5 min) in the presence of 50 μM ascorbate (red). (c) *I*–*V* curves of a graphene device reconnected by control DNA, after DNA connection (black) and after further Cu^2+^ treatment for 5 min (red) (0.5 nM with 50 μM ascorbate).

Under optimized conditions, the maximum connection yield for the DNAzyme molecules was found to be ∼27%, which corresponds to the cutting yield of ∼36%.^51^ On the basis of these data, the analysis of the number of junctions that contribute to the charge transport, using the binomial distribution, demonstrates that in most cases only one or two junctions contribute to the charge transport of the devices.^52^ Fig. 2a shows the comparison of the *I*–*V* curves of a representative DNAzyme-reconnected device before and after cutting. In brief, the black curve shows the S–D current (*I*_D_) plotted against the gate voltage (*V*_G_) at constant S–D bias voltage (*V*_D_ = –50 mV) before cutting. The red curve, taken after cutting, shows no conductance down to the noise limit of the measurement (≤100 fA) due to the nanogaps. After molecular connection, we observed the recovery of the original property, albeit at reduced current values (black trace in Fig. 2b). These observations are consistent with our previous cases.^39^ Interestingly, upon addition of 0.5 nM Cu^2+^, in the presence of 50 μM ascorbate in HEPES buffer (25 mM, pH 7.0, 750 mM NaCl), the device conductance decreased down to zero (red trace in Fig. 2b) (27 out of 29 devices tested). This is attributable to a Cu^2+^-catalyzed cleavage of the substrate strand, resulting in a gap between the graphene–molecule junction. We found that the presence of ascorbate is necessary to significantly enhance the reaction rate (Fig. S2†), similar to those observed previously.^34–36^ Such an enhancement has been ascribed to ascorbate reduction of Cu^2+^ to form Cu^+^, which subsequently reacted with oxygen to accelerate the oxidative cleavage of DNA.^21,34–36^

To eliminate potential artifacts due to the addition of an electrolyte solution, a control experiment was conducted using 50 μM ascorbate solution without Cu^2+^ and no obvious conductance changes were observed under the same conditions (Fig. S3†). In addition, to confirm that the observed signal is indeed due to the presence of the Cu^2+^-specific DNAzyme, we used a control DNA without the Cu^2+^-dependent catalytic cleavage site to connect the graphene gaps (see the ESI†). In the presence of 0.5 nM Cu^2+^, no obvious change in conductance was observed (Fig. 2c). These results highlight the unique role of specific DNAzyme sequences in copper detection.

**Fig. 3:**
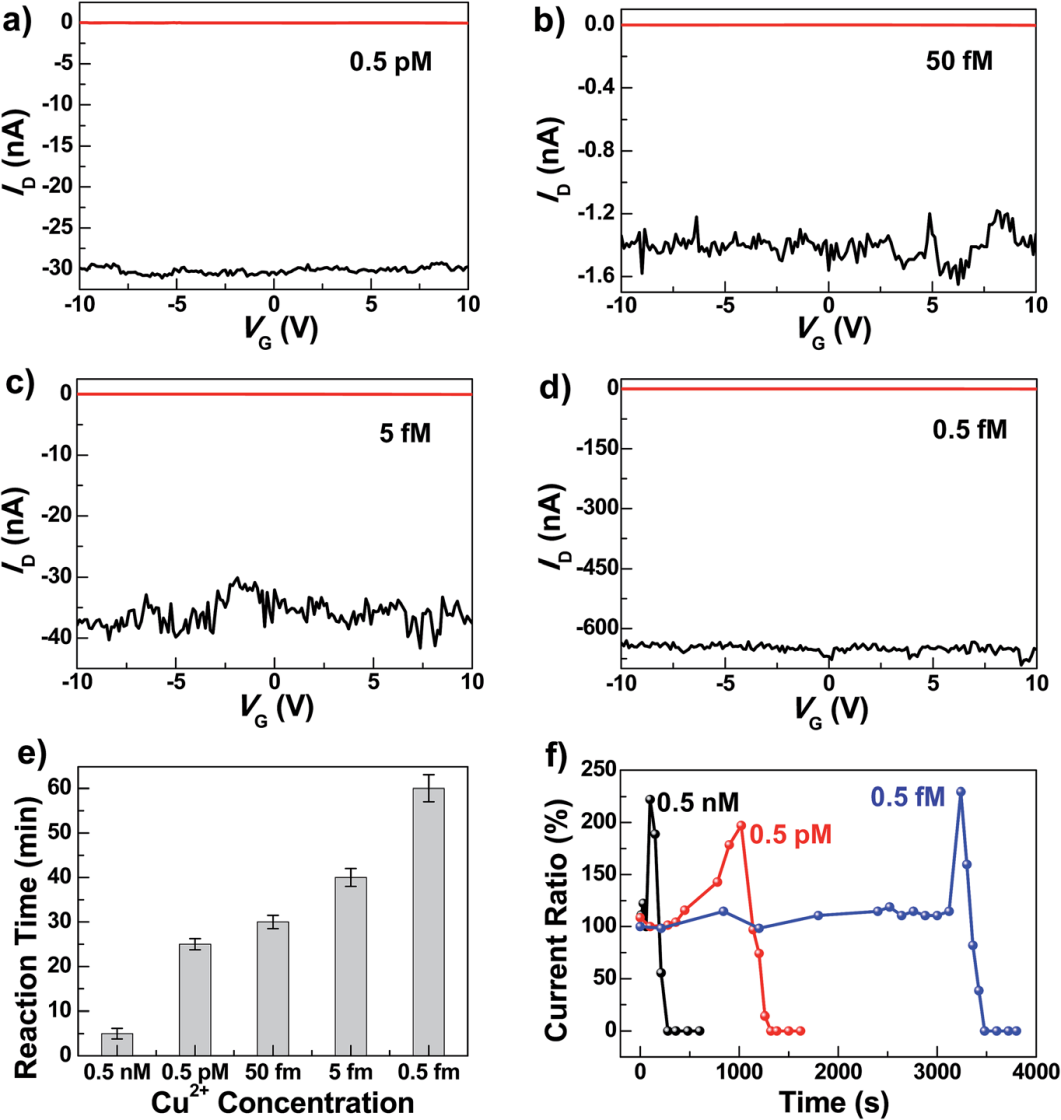
(a–d) Device characteristics of different graphene–DNAzyme junctions after DNA connection (black) and further Cu^2+^ treatments of different concentrations in the presence of 50 μM ascorbate (red) (a: 0.5 pM; b: 50 fM; c: 5 fM; d: 0.5 fM). (e) Statistical reaction times for the complete Cu^2+^ catalytic reactions at different concentrations. (f) Concentration-dependent dynamics of the Cu^2+^ catalytic reactions. All the measurements were performed at *V*_D_ = –50 mV.

To test the sensitivity of these graphene–DNAzyme junctions, we investigated the responses of newly-prepared DNAzyme-rejoined devices to Cu^2+^ in different concentrations (0.5 nM, 0.5 pM, 0.05 pM, 5 fM, and 0.5 fM) (Fig. 3). Even though the *I*_D_ values vary depending on the device fabrications, the reaction time is not affected, because it is characteristic of the Cu^2+^-induced cleavage of the DNAzyme. As shown in Fig. 3e, the reaction time is longer when the Cu^2+^ concentration is lower. For example, we observed the device breakage in less than 5 min for 0.5 nM Cu^2+^ compared to ∼60 min for 0.5 fM Cu^2+^. The detailed dynamics of the cleavage process were measured by monitoring the current change ratios as a function of time at different concentrations (Fig. 3f). We found that the diffusion and binding of metal ions are concentration-dependent while the rate of the breakage is similar, completed within ∼240 s. The time-dependent sensing behaviors can be explained as follows. For a chemical reaction *n*A + *m*B → *p*P + *q*Q, the reaction rate obeys the following kinetic formula: *r* = *K*_T_ *C*_A_ *C*_B_, where *K* is the rate constant and *C* is the concentration of each reactant. Since our device consists of only one or a few DNA molecules spanning the nanogaps, the DNA concentration can be considered constant for this reaction. Furthermore, the rate of the binding reaction between DNA and metal ions is directly proportional to the concentration of the metal ions although the actual diffusion rate doesn't change. If the concentration of Cu^2+^ is lower, *r* is smaller, therefore resulting in the longer reaction time. Conversely, the fact that the breakage process after binding didn't show concentration dependence demonstrates the single-event sensitivity, which is of crucial importance to future single-molecule biodetection. The current increase we observed before DNA cleavage is attributable to the rigidification of DNA conformation during the initial metal binding, improving π–π stacking between base pairs and thus increasing the DNA conductivity.^47,49^ After the Cu^2+^ binding and conformational change, the Cu^2+^-promoted DNAzyme cleavage resulted in gaps between the two electrodes and thus the gradual decrease of the current down to zero. Remarkably, the Cu^2+^ in all concentrations investigated resulted in the complete breakdown of the drain current, even at 0.5 fM, although the conductance values varied from device to device. This detection limit is significantly lower than those of previously reported Cu^2+^ sensors, such as the lateral flow nucleic acid biosensors (10 nM),^53^ phosphorescence sensor (35 nM),^14^ optical chemosensors (10 nM),^54^ ratiometric fluorescence sensors (3 μM),^20^ and DNAzyme catalytic beacon sensors, which represent one of the most sensitive turn-on Cu^2+^ sensors (0.6 nM).^21,55^ The realization of atomic level precision in the cutting procedure and precise control of the molecular conformation on the substrate within the graphene gaps and the contact configuration are challenges for future studies to overcome.

**Fig. 4:**
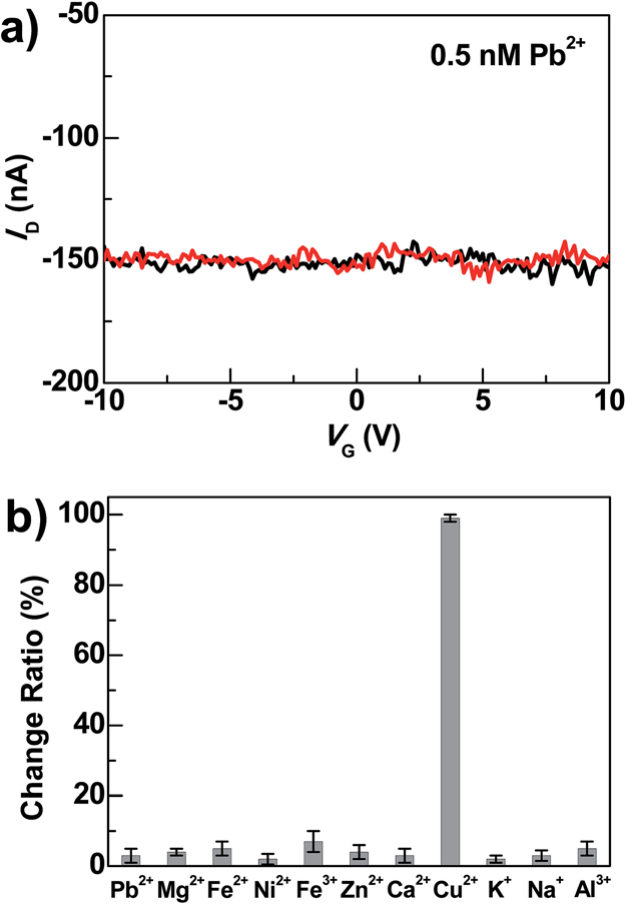
(a) *I*–*V* curves of DNAzyme-bridged graphene devices after DNAzyme connection (black) and after further treatments with Pb^2+^ (∼0.5 nM) under the same conditions in the presence of 50 μM ascorbate (red). (b) Statistical comparisons of conductance changes under the same conditions in the presence of 50 μM ascorbate (0.5 nM of Pb^2+^, Mg^2+^, Fe^2+^, Ni^2+^, Fe^3+^, Zn^2+^, Ca^2+^ and Cu^2+^; 5 mM of K^+^; 135 mM of Na^+^; 60 nM of Al^3+^). All the measurements were performed at *V*_D_ = –50 mV.

Beside high sensitivity, high selectivity is also crucial for sensing. To evaluate the selectivity of DNAzyme-bridged graphene devices, we measured the conductance changes of freshly prepared working devices after adding Pb^2+^ (0.5 nM), Zn^2+^ (0.5 nM), Mg^2+^ (0.5 nM), Ca^2+^ (0.5 nM), Fe^2+^ (0.5 nM), Fe^3+^ (0.5 nM), Ni^2+^ (0.5 nM), K^+^ (5 mM), Na^+^ (135 mM) and Al^3+^ (60 nM) for 5 min under the same conditions. As illustrated in Fig. 4 and S4,† the responses of the devices to these metal ions were essentially unchanged in the presence or absence of these metal ions. In contrast, further treatment of the above systems with 0.5 nM Cu^2+^ in the presence of 50 μM ascorbate for 5 min resulted in the loss of device conductance. Therefore, these results demonstrate excellent selectivity of these DNAzyme-functionalized devices towards Cu^2+^.

## Conclusions

In summary, we have demonstrated a platform of using single-molecule graphene–DNAzyme junctions to achieve direct electrical detection of paramagnetic Cu^2+^ with ultra-high femtomolar sensitivity and high selectivity. While most metal ion sensors reported previously relied on optical properties that require the labeling of DNAzymes with either fluoresencent or colorimetric groups, and these optical properties can often be interfered by background fluorescence or colors, the current system integrated the DNAzymes directly into electrical circuits without labeling them, and with electrical signals that are much less vulnerable to interference. Even though several examples of DNAzyme-based metal ion sensors have been published, none of the reported DNAzyme sensors have achieved ultra-high sensitivity as reported in this work. We accomplished this task for using a novel signal transduction mechanism using a graphene–DNAzyme junction. Just like organic molecule-based metal ion sensors, despite numerous papers published in the field for many years, there is still a major advance possible for better performance if a new signal transduction mechanism can be introduced. Since DNAzymes selective for a variety of metal ions can be obtained through *in vitro* selection, the sensing system demonstrated here can be applied to the detection of many other metal ions. Finally, since the graphene devices are compatible to current complementary metal oxide semiconductor (CMOS) technologies, our system has the potential for the development of direct, low-cost, high-throughput and real-time detection arrays for chemical and biological reactions.
