# Supplementary material for: Graphene–DNAzyme junctions: a platform for direct metal ion detection with ultrahigh sensitivity
Source: Chem Sci. 2015 Feb 5;6(4):2469–73. doi: 10.1039/c4sc03612c (PMC4583199; doi:10.1039/c4sc03612c)
Supplement: Supplementary file 1 [file SC-006-C4SC03612C-s001.pdf]

Electronic Supplementary Material (ESI) for Chemical Science.
This journal is © The Royal Society of Chemistry 2015

ELECTRONIC SUPPLEMENTARY INFORMATION FOR

Graphene-DNAzyme Junctions: A Platform for Direct Metal Ion Detection with Ultrahigh Sensitivity

Li Gao,[‡a] Le-Le Li,[‡b] Xiaolong Wang,[a] Peiwen Wu,[b] Yang Cao,[a] Bo Liang,[c] Xin Li,[c] Yuanwei Lin,[a] Yi Lu,*[b] and Xuefeng Guo*[ad]

[a]Center for Nanochemistry, Beijing National Laboratory for Molecular Sciences, State Key Laboratory for Structural Chemistry of Unstable and Stable Species, College of Chemistry and Molecular Engineering, Peking University, Beijing 100871, P. R. China. Email: guoxf@pku.edu.cn
[b]Department of Chemistry, University of Illinois at Urbana-Champaign, Urbana, Illinois 61801, USA. Email: yi-lu@illinois.edu
[c]Adesso Advanced Materials Wuxi Co., Ltd., Huihong Industrial Park, 18 Xishi Road, New District, Wuxi, Jiangsu Province, 214000, P. R. China.
[d]Department of Materials Science and Engineering, College of Engineering, Peking University, Beijing 100871, P. R. China.
‡These authors contributed equally.

Table of Contents

1 **Materials** S2
2 **Device Fabrication and chemical treatments** S2
3 **DNA Cleavage confirmation** S3
4 **Control Experiments** S4
5 **Summary for molecular conductance** S6
6 **References** S8

1. Materials

N-(3-Dimethylaminopropyl)-N'-ethylcarbodiimide hydrochloride (EDCI) and N-Hydroxysulfosuccinimide sodium salt (Sulfo-NHS) were purchased from Sigma-Aldrich, Inc. Ultra pure water with an electrical resistivity larger than 18.2 MΩ cm was used throughout. All oligonucleotides were synthesized by Takara Biotechnology Co., Ltd. (Dalian, China). The oligonucleotide sequences of Cu^{2+} DNAzyme and control DNA are listed as follows:

Cu^{2+} DNAzyme:

H_2N-$(CH_2)_3$-Pi-5'-TTCTTTCTAATACGGCTTACC-3'-Pi-$(CH_2)_3$-NH_2

5'-GGTAAGCCTGGGCCTCTTTCTTTTTAAGAAAGAAC-3'

Control DNA:

H_2N-$(CH_2)_3$-Pi-5'-TTCTTTCTAATACGGCTTACC-3'-Pi-$(CH_2)_3$-NH_2

5'-GGTAAGCCTGGGCCTCTTTCTTTTTAACAAACAAC-3'

2. Device Fabrication and chemical treatments

Device Fabrication: The cut graphene devices were fabricated by a new dash-line lithographic (DLL) method described in detail elsewhere.[1,2] In brief, we designed a DesignCAD file with a 5 nm-width dash line to open an indented window in a spin-cast layer of polymethylmethacrylate (PMMA) by using ultrahigh-resolution e-beam lithography. The graphene sheet was then locally cut through the open window via oxygen plasma ion etching. By exploiting the gradual etching and undercutting of PMMA, we achieved narrow gaps between indented graphene point contacts. Then, we used the strategy developed previously to graft DNAs into the gaps.[3,4] The cut SLG devices were immersed in a 50 mM MES buffer solution (pH 4.7) containing 10 mM amide coupling agent (Sulfo-NHS) and 5 mM activating agents (EDCI) for 12 hours. After removing from the solution, washing with copious buffer solution, and drying with nitrogen gas, the activated SLG termini were reacted with 10 μM amine-modified DNA dissolved in phosphate buffered saline solution (pH 7.2) to covalently bridge the gaps. After 24 hours of reaction, the devices were taken out from the solution, washed with H_2O, and then dried with nitrogen gas.

Chemical treatments: For Cu^{2+} treatment experiments, the newly rejoined devices were immersed in the buffer solution (750 mM NaCl, 25 mM HEPES, pH 7.0) with or without 50 μM ascorbate. After incubation, the devices were removed from the solution, washed with fresh copious buffer solution, and dried with nitrogen gas. Other experiments using different metal ions, such as Pb^{2+}, Ni^{2+}, Zn^{2+}, Fe^{2+}, Fe^{3+}, Mg^{2+}, and Ca^{2+}, are the same as the Cu^{2+} process. The device characterization was carefully carried out at room temperature in the ambient atmosphere using an Agilent 4155C semiconductor characterization system (DC measurements) and a Karl Suss (PM5) manual probe station with consistent moisture content (30%).

3. DNA Cleavage confirmation

Gel-based assays were carried out to confirm the cleavage reaction before the DNA reconnection: 1 μM of FAM-labeled Cu-Sub and 2 μM of Cu-Enzyme (Apt-con) were annealed in 750 mM NaCl, 25 mM HEPES, pH 7.0. After annealing, 50 μM of ascorbate was also added to increase the cleavage rate. Before addition of Cu^{2+}, a 10 μl aliquot was taken out as the zero time point (lane a for Apt-con, lane b for Cu-Enzyme). After adding Cu^{2+}, 10 μl aliquots were taken out at designated time points and were quenched in a stop buffer containing 8 M urea, 50 mM EDTA, 0.05% xylene cyanol, and 50 mM Tris acetate, pH 8.2. The samples were loaded into 20% polyacrylamide gel electrophoresis to separate cleaved and uncleaved substrates. The gel was imaged with a fluorescence scanner (FLA-3000G, Fuji) by exciting at 450 nm and setting the emission filter at 520 nm. Lane c to d contained the Apt-con and Cu-Enzyme 25 min after Cu^{2+} addition, respectively. Cleavage was only observed after addition of Cu^{2+} to the Cu-Enzyme. These control experiments form a good basis for our next experiments. .

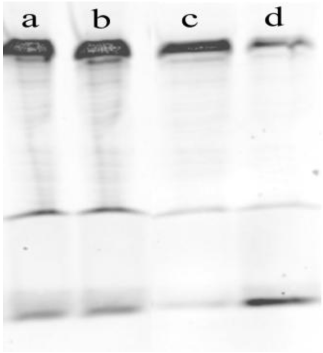

Figure S1. Electrophoresis for treated DNA sequences

4. Control Experiments

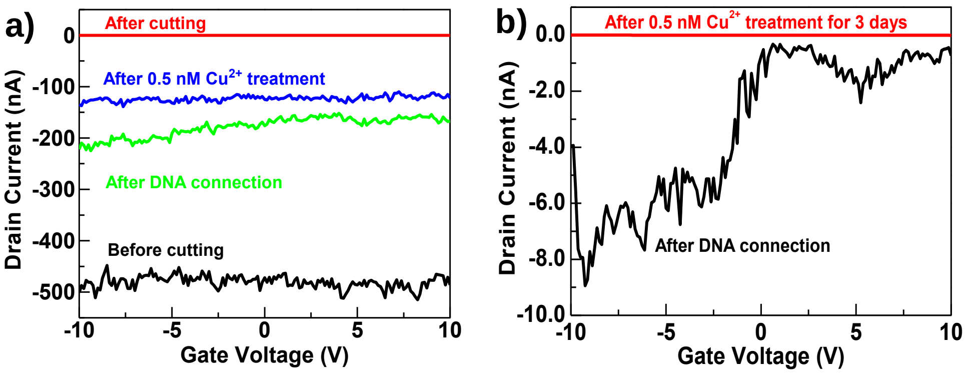

Figure S2. (a) *I–V* curves of a DNAzyme-reconnected device before cutting (black), after cutting (red), after DNA connection (green), and after further Cu^{2+} treatments without ascorbate for 5 minutes (blue). (b) *I–V* curves of another DNAzyme-reconnected device after DNA connection (black) and after further Cu^{2+} treatments without ascorbate (red). $V_D = -50$ mV.

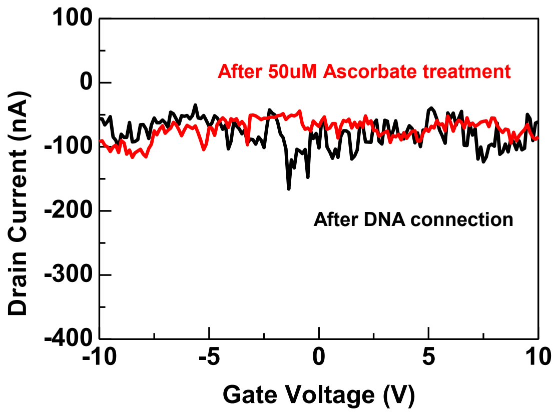

Figure S3. *I-V* curves of a DNAzyme-reconnected device after DNA connection (black) and after further ascorbate treatment (red). Control experiments using only 50 μM ascorbate treatment did not lead to the obvious conductance changes at the same conditions. $V_D = -50$ mV.

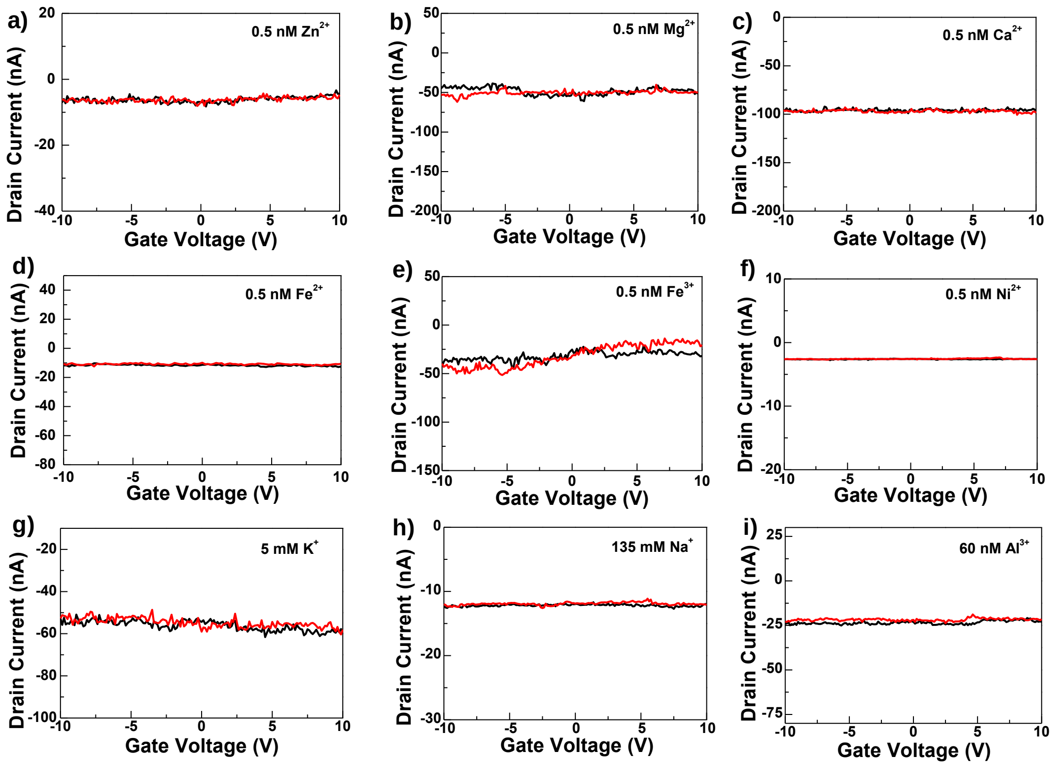

Figure S4. The conductance comparisons of different DNAzyme-bridged graphene devices after DNAzyme connection (black) and after further treatments with different metal ions (~0.5 nM) under the same conditions in the presence of 50 μM ascorbate (red) (0.5 nM of Pb^{2+}, Mg^{2+}, Fe^{2+}, Ni^{2+}, Fe^{3+}, Zn^{2+}, Ca^{2+} and Cu^{2+}; 5 mM of K^{+}; 135 mM of Na^{+}; 60 nM of Al^{3+}). The concentrations of K^{+}, Na^{+} and Al^{3+} are close to their values in human body. All the measurements were performed at V_D = –50 mV.

5. Summary for molecular conductance

Table 1: Summary of the resistance values of different rejoined devices for sensitivities before cutting, and after reconnection.

	Before Cutting	After Reconnection	
Device number	Effective Resistance[a] (Ω)	Effective Resistance[a] (KΩ)	Molecular Conductance (e^2/h)
A	31.85×10^{3}	625	4.37×10^{-2}
B	2.29×10^{4}	1785.71	1.47×10^{-2}
C	17.73×10^{3}	1650.17	1.59×10^{-2}
D	4.35×10^{3}	35714.29	7.27×10^{-4}
E	7.78×10^{3}	1388.89	1.88×10^{-2}
F	3.94×10^{3}	76.92	0.35

[a]Resistance values were calculated using a gate bias of −40 V and a source-drain bias of −50 mV before cutting and after reconnection.

Table 2: Summary of the resistance values of different rejoined devices for selections before cutting, and after reconnection.

	Before Cutting	After Reconnection	
Device number	Effective Resistance[a] (Ω)	Effective Resistance[a] (KΩ)	Molecular Conductance (e^2/h)
A	1.31×10^{3}	329.82	7.85×10^{-2}
B	3.22×10^{3}	19008.2	1.36×10^{-3}
C	32.26×10^{3}	8301.08	3.12×10^{-3}
D	47.17×10^{3}	1000	2.72×10^{-2}
E	1.52×10^{3}	1470.59	1.76×10^{-2}
F	12.25×10^{3}	3559.17	7.28×10^{-3}
G	28.90×10^{3}	520.83	5.27×10^{-2}
H	3.73×10^{4}	4098.36	6.38×10^{-3}
I	4.27×10^{3}	891.27	2.92×10^{-2}
J	4.13×10^{3}	2136.75	1.21×10^{-2}

[a]Resistance values were calculated using a gate bias of −40 V and a source-drain bias of −50mV before cutting and after reconnection.

References:

(1) Y. Cao, S. Dong, S. Liu, L. He, L. Gan, X. Yu, M. L. Steigerwald, X. Wu, Z. Liu and X. Guo, *Angew. Chem. Int. Ed*., 2012, **51**, 12228.

(2) Y. Cao, S. Dong, S. Liu, Z. Liu and X. Guo, *Angew. Chem. Int. Ed.*, 2013, **52**, 3906.

(3) X. Guo, A. A. Gorodetsky, J. Hone, J. K. Barton and C. Nuckolls, *Nat. Nanotechnol*., 2008, **3**, 163.

(4) S. Liu, X. Zhang, W. Luo, Z. Wang, X. Guo, M. L. Steigerwald and X. Fang, *Angew. Chem. Int. Ed.*, 2011, **50**, 2496.
